# Supplementary material for: Hippocampal inactivation during rearing on hind legs impairs spatial memory
Source: Sci Rep. 2023 Apr 15;13:6136. doi: 10.1038/s41598-023-33209-9 (PMC10105745; doi:10.1038/s41598-023-33209-9)
Supplement: Supplementary file 1 — Supplementary Information 1. [file 41598_2023_33209_MOESM1_ESM.pdf]

Manuscript title: Hippocampal inactivation during rearing on hind legs impairs spatial memory
Authors: Dylan Layfield, Nathan Sidell, Kevin Blankenberger, Ehren Lee Newman

Supplemental materials and supplemental captions

Video S1 – Demonstration 1 of automatic detection of rearing events by the 3D camera system. Two panels are shown. The left panel shows the RBG video capture from the Intel RealSense D435 camera as a rat completes the study phase. The right panel shows a thresholded version of the same capture showing blobs that the 3D camera detects as crossing the height threshold used to detect rears. A large green circle is superimposed and marks the boundary of the region of interest. Any blobs detected outside of this circle are ignored. Blobs classified as rearing events based on crossing the height threshold inside the region of interest and having a sufficiently large area are marked with red circles. This video was produced for demonstration purposes. The rat was not implanted with optical fibers nor connected to the laser.

Video S2 – Demonstration 2 of automatic detection of rearing events by the 3D camera system. The only difference is that all eight arm doors were opened. See caption for Video S1 for description.

Experimental Group Rats

	Control	Rear	Delay
Subject 1	15	10	7
Subject 2	5	11	6
Subject 3	16	13	8
Subject 4	10	9	7
Subject 5	17	7	6
Subject 6	17	9	8

Table S1 – Number of trials each rat contributed to each experimental condition for rats in the experimental (opsin + reporter) group.

Control Group Rats

	Control	Rear	Delay
Subject 1	12	12	15
Subject 2	6	6	7
Subject 3	6	8	6
Subject 4	13	14	13
Subject 5	15	14	11
Subject 6	11	9	12
Subject 7	10	8	12

Table S2 - Number of trials each rat contributed to each experimental condition for rats in the control (reporter but no opsin) group.

Experimental Group Rats

		Number of reentries	Rotational Bias	Max sequence length	Number of opposite entries
Off	Mean +/- std. err	0.000 +/- 0.000	0.563 +/- 0.089	0.575 +/- 0.076	0.713 +/- 0.082
Rear	Mean +/- std. err	0.034 +/- 0.024	0.864 +/- 0.118	0.695 +/- 0.095	0.746 +/- 0.099
Delay	Mean +/- std. err	0.000 +/- 0.000	0.595 +/- 0.115	0.524 +/- 0.099	0.952 +/- 0.162

Table S3 – Analyses of behavioral strategy and error types in the test phase for rats in the experimental (opsin and reporter) group. Shown are mean and standard error over rats for four behaviors: 1) Number of reentries = count of the number of times a rat leaves an arm and then reenters the same arm prior to entering other arms within a test phase. 2) Rotational Bias = an examination of entries into arms immediately adjacent to the arm the rat leaves that quantifies how often they turn one direction versus the other. It is calculated as the absolute value of the difference in the number of clockwise and counterclockwise transitions. It ranges from zero to the number of total arm entries. A value of zero indicates a balanced likelihood of transitioning one way versus the other. Values above zero indicate how many more times the rat transitioned in one direction relative to the other. 3) Max sequence length = a quantification of the maximum number of consecutive transitions to adjacent arms in one direction or the other. It can range in value from zero, for never transitioning to adjacent arms, to greater values up to the total arm entries if the rat progressed from one arm to the next around the maze. 4) Number of opposite entries = count of the number of times a rat leaves an arm and then enters the arm immediately across from the exited arm in a test phase.

Control Group Rats

		Number of reentries	Rotational Bias	Max sequence length	Number of opposite entries
Off	Mean +/- std. err	0.014 +/- 0.014	0.411 +/- 0.065	0.397 +/- 0.061	0.808 +/- 0.089
Rear	Mean +/- std. err	0.027 +/- 0.019	0.603 +/- 0.080	0.521 +/- 0.063	0.863 +/- 0.108
Delay	Mean +/- std. err	0.040 +/- 0.023	0.480 +/- 0.088	0.387 +/- 0.063	0.867 +/- 0.084

Table S4 – Analyses of behavioral strategy and error types in the test phase for rats in the control (reporter but no opsin) group. See Table S3 for condition label definitions.
